# Factors obstetricians, family physicians and midwives consider when counselling women about a trial of labour after caesarean and planned repeat caesarean: a qualitative descriptive study

**DOI:** 10.1186/s12884-020-03052-1

**Published:** 2020-06-18

**Authors:** Christine Kurtz Landy, Wendy Sword, Jackie Cramp Kathnelson, Sarah McDonald, Anne Biringer, Maureen Heaman, Pam Angle

**Affiliations:** 1 Faculty of Health, School of Nursing, York University, HNES 312A, 4700 Keele Street, Toronto, Ontario M3J 1P3 Canada; 2 McMaster University, 1280 Main Street West, Hamilton, Ontario L8S 4L8 Canada; 3 Faculty of Health, York University, HNES 312A, 4700 Keele Street, Toronto, Ontario M3J 1P3 Canada; 4 Department of Obstetrics and Gynecology, McMaster University, 1280 Main Street West, Hamilton, Ontario L8S 4K1 Canada; 5 Department of Family and Community Medicine, University of Toronto, Ada Slaight and Slaight Family director of Family Medicine Maternity Care, Toronto, Canada; 6 Ray D Wolfe Department of Family Medicine, Sinai Health System, 60 Murray St, Toronto, Ontario M5T 1L9 Canada; 7 College of Nursing, Rady Faculty of Health Sciences, Helen Glass Centre for Nursing, University of Manitoba, 89 Curry Place, Winnipeg, MB R3T 2N2 Canada; 8 Sunnybrook Research Institute, 2075 Bayview Ave, Toronto, ON M4N 3M5 Canada

## Abstract

**Background:**

Repeat caesarean sections (CSs) are major contributors to the high rate of CS in Canada and globally. Women’s decisions to have a planned repeat CS (PRCS) or a trial of labour after CS (TOLAC) are influenced by their maternity care providers. This study explored factors maternity care providers consider when counselling pregnant women with a previous CS, eligible for a TOLAC, about delivery method.

**Methods:**

A qualitative descriptive design was implemented. Semi-structured, one-to-one in-depth telephone interviews were conducted with 39 maternity care providers in Ontario, Canada. Participants were recruited at 2 maternity care conferences and with the use of snowball sampling. Interviews were audio recorded and transcribed verbatim. Data were uploaded into the data management software, NVIVO 10.0 and analyzed using qualitative content analysis.

**Results:**

Participants consisted of 12 obstetricians, 13 family physicians and 14 midwives. Emergent themes, reflecting the factors maternity care providers considered when counselling on mode of delivery, were organized under the categories clinical/patient factors, health system factors and provider preferences. Maternity care providers considered clinical/patient factors, including *women’s choice … with conditions, their assessment of women’s chances of a successful TOLAC, their perception of women’s risk tolerance, women’s preferred delivery method, and their perception of women’s beliefs and attitudes about childbirth*. Additionally, providers considered health system factors which included *colleague support for TOLAC* and *time needed to mount an emergency CS*. Finally, provider factors emerged as considerations when counselling. They included *provider preference for PRCS or TOLAC*, *provider scope of practice*, *financial incentives and convenience related to PRCS, past experiences with TOLAC and PRCS and providers’ perspectives on risk of TOLAC.*

**Conclusion:**

The findings highlight the multiplicity of factors maternity care providers consider when counselling women. Effectively addressing clinical, health care system and personal factors that influence counselling may help decrease non-medically indicated PRCS.

## Background

There is global concern about the high rates of caesarean section (CS) across middle and high income countries [1]. The average CS rate for the Organization for Economic Co-operation and Development (OECD) countries rose from 20.0% in 2000 to 28.1% in 2017 and varied significantly across countries from as low as 14.8 to 16.6% in Israel and the European Nordic countries, 28.8% in Canada, 32.0% in the USA, and as high as 53.1% in Turkey [1, 2]. A significant factor contributing to rising CS rates is the increase in planned repeat caesarean section (PRCS) [1, 3]. Repeat CS rates vary across OECD countries, ranging from 45.5 to 93.5% across Europe [4], and is 86.7% in the United States [5] and 81.3% in Canada [2]. One strategy recommended to decrease CS rates is to increase trial of labour after caesarean (TOLAC) rates among eligible women [6, 7]. The best available evidence suggests that vaginal birth after one low segment transverse CS is a safe and reasonable choice for most women and is associated with lower maternal mortality, less morbidity for mothers and infants [8–11] and decreased cost [12]. Findings from a large systematic review on vaginal birth after caesarean section (VBAC) indicate that although there has been a significant decrease in VBAC rates, the VBAC success rate and associated adverse outcomes have not changed [8]. This finding suggests that the reduction in women undergoing TOLAC does not reflect improved selection of patients for VBAC [3, 13]. Success rates for TOLAC range from 28 to 85% [3, 14–16] and are higher for women with a previous CS for nonrecurring indications, such as breech presentation, and for women with a previous vaginal birth [17–20]. Furthermore, TOLAC is supported by obstetrical best practice guidelines in numerous countries [7, 9, 11, 21].

As the majority of women with one previous low segment CS are eligible for a TOLAC [3], the high rates of repeat CS cannot be explained by medical indications alone. Factors, such as women’s preferences, maternity care providers’ counselling, attitudes and beliefs about the safety of PRCS and TOLAC, and systems factors such as hospital support for TOLAC influence PRCS rates [3, 22–24].

Maternity care providers play a key role in advising and influencing women’s decision regarding delivery method [21, 22, 25–28]. Findings from a systematic review and meta-synthesis indicate that providers’ beliefs and attitudes are among the primary factors influencing provider decision making for CS and TOLAC [22]. Moreover, there is international variation in maternity care providers’ beliefs and attitudes about TOLAC and/or PRCS for eligible women [22, 29–32]. Cox and colleagues [31], in a study undertaken in Florida, USA, found that obstetricians and midwives avoided TOLAC for convenience and fear of liability. In the UK, providers attitudes about TOLAC have been mixed. Sur and colleagues [33] reported that UK obstetricians advised women to choose the option they, themselves preferred, whereas, Kamal and colleagues [32] found that UK obstetricians and midwives supported TOLAC. In Nordic countries, providers are reported to have a strong belief in natural childbirth and support TOLAC as the first choice for eligible women [22, 29].

To date little is known about the factors Canadian maternity care providers consider when counselling pregnant women, eligible for a TOLAC, on whether to choose a TOLAC or PRCS. The Society of Obstetricians and Gynecologists of Canada (SOGC) recommends a TOLAC be offered to all women with one previous low-segment transverse CS who have no contraindications [21]. Although the majority of Canadian women with one previous CS are eligible for TOLAC, less than one third plan a TOLAC [34]. Two Canadian studies that examined maternity care providers’ attitudes regarding intrapartum care found that provider age and whether the provider practiced both antepartum and intrapartum care, or antepartum care alone, affected attitudes towards the use of CS [35, 36]. A third Canadian study, in which a small sample of 16 obstetricians, midwives and family doctors were interviewed on barriers to accessing TOLAC, found that physicians had limited discussion with patients about patient preferences, but provided information about the risks and benefits of TOLAC and PRCS [37]. As maternity care providers play an important role in advising women on method of delivery, insight into the factors they consider when counselling is important to the development of strategies to increase the uptake of TOLAC in Canada.

The objective of our study was to explore the clinical/patient, health care system and provider factors that influence obstetricians, family physicians, and midwives when counselling pregnant women with one previous CS, who are eligible for a TOLAC, about TOLAC and PRCS. Eligibility for TOLAC was based on the criteria outlined in the Society of Obstetricians and Gynecologists of Canada (SOGC) vaginal birth after caesarean section guidelines [9].

## Methods

This qualitative descriptive study is the first phase of a sequential mixed methods study that examined the factors maternity care providers consider when counselling women regarding TOLAC and PRCS. We adhered to Sandelowski’s [38] principles of fundamental qualitative description to guide our sampling, data collection, and analysis. Qualitative description allows for comprehensive exploration and description of a phenomenon, and is appropriate when little is known about a phenomenon under investigation [38].

### Setting

The study took place in the province of Ontario, Canada. Ontario has the highest yearly number of births in Canada (143,574 of a total 387,516 in 2015/2016) [39] and among the highest overall and repeat CS rates (28.4 and 81.3% respectively) [2]. There are large regional variations in CS rates across the province. In 2016–2017, primary and repeat CS rates ranged from 15.5 to 22.0%, and 71.5 to 86.6% respectively across regions [2]. From 2016 to 2018, Ontario obstetricians, family physicians, and midwives attended approximately 76.3, 7.4, and 10.7% of births respectively, including TOLACs; 5.6% were attended by other [40]. Generally, only obstetricians and surgeons perform CSs.

### Participants and recruitment

A purposeful sample of maternity care providers was recruited. We estimated a total sample of 40 maternity care providers would suffice to reach data saturation [41]. Inclusion criteria were: 1) licensed obstetrician, family physician or midwife; 2) provided prenatal and/or intrapartum care to pregnant women with a previous CS; and 3) could read, write and speak English. Maximum variation sampling [41] guided recruitment of a broad range of perspectives about factors maternity care providers consider when counselling pregnant women eligible for a TOLAC about delivery method. Participants were selected to ensure diversity in: 1) professional credentials, i.e., obstetricians, family physicians, and midwives; 2) demographic characteristics such as gender and age; 3) years in practice; and 4) rural and urban practice settings.

Participants were recruited at two Ontario maternity care provider conferences where a booth was set up to promote our study. Providers who expressed interest were given information about the study and provided their email addresses if they wished to participate. In order to ensure we had participants from rural communities, we sent invitation letters to maternity care providers working at three primary care centres in northern and rural Ontario communities. Additionally, snowball sampling was employed, i.e., research team members identified potential participants from within their professional networks. Information about the study was emailed to these potential participants. Those interested in participating in the study were asked to contact the research coordinator by email or telephone to set up a time for an interview. Recruitment continued until we achieved our maximum variation sample and data saturation.

### Data collection

Participants completed one in-depth 30 to 60-min semi-structured telephone interview. At the beginning of the interview the study was explained, and verbal informed consent was obtained. The semi-structured interview guide was developed by the research team and was informed by the literature [3, 22]. It focused on exploring factors consider when counselling pregnant women, with one previous CS and eligible for a TOLAC, about TOLAC and PRCS (See sample interview questions in Table 1). Participants completed a demographic questionnaire and were emailed a $25 gift card in appreciation for their participation. The research coordinator, experienced in qualitative interviewing, conducted the majority of interviews. A graduate student who was an experienced obstetrical nurse and trained by CKL completed five interviews. Data collection and analysis were undertaken concurrently. At the end of each interview, a detailed summary of the interview was created that identified emerging themes that were immediately apparent in the data and those requiring further exploration in.

**Table 1:** Sample Interview Questions

1. When you discuss delivery method with pregnant women who have had a previous caesarean section, eligible for a TOLAC, what kinds of factors do you consider when counselling them?	
2. In your experience, what factors do you believe are most important to women in their decision regarding preference of delivery method?	
3. Under what circumstances do you usually recommend a trial of labour?	
4. Under what circumstances do you usually recommend a planned repeat caesarean section?	

subsequent interviews. All interviews were audio recorded, transcribed verbatim, and checked for accuracy.

### Data analysis

Conventional qualitative content analysis, in which coding categories are derived directly from the text, was used to analyze the data [42]. Initially, specific words and phrases that described factors maternity care providers considered when counselling were coded. Memos were made while coding to link emergent impressions and thoughts, and to help make inferences from the data [42, 43]. Codes representing similar ideas or patterns within and across interviews were clustered into categories. Data management and coding were done using NVIVO 10.0 [44].

Early in the data analysis process, research team members individually reviewed and coded two transcripts (CKL, WS, JC) and then met to examine consistency of coding. Discrepancies in coding were discussed until consensus was reached. A preliminary coding scheme was developed and guided analysis of subsequent interview transcripts. The analytic strategy of constant comparison was used to identify similarities and differences in factors providers considered when counselling [45]. The research coordinator (JC) coded the interviews and met weekly with CKL throughout the coding process to discuss themes emerging from the data and revise the coding scheme as necessary. Peer debriefing was undertaken during data analysis with a colleague with expertise in qualitative methods to clarify interpretation of data [46]. When our data analysis indicated data saturation, the research team members (SM, AB, MM, MH, PA, HM) who had experience in maternity care, but were not involved in data collection or analysis, reviewed the emergent themes. The purpose was to determine whether there were potential provider counselling factors that were not captured by our data, which would have suggested that further recruitment of participants was required [46].

An audit trail of all decisions about data collection and analysis was maintained throughout the research process. All categories and themes were firmly grounded in the data and memos about coding decisions were kept, along with copies of coding schemes as they evolved. The interdisciplinary composition of the research team, with diverse backgrounds in research and/or maternity care, contributed to data credibility and dependability [47].

The study was approved by the York University Research Ethics Board and Hamilton Integrated Research Ethics Board. Participants were identified with a code signifying provider type (obstetrician - OB, Family physician - FP, midwife - MW) and a number to allow for identification of interview text from specific interviews and ensure participant confidentiality.

## Results

### Demographic characteristics

Thirty-nine maternity care providers completed the in-depth interviews. The sample included 12 obstetricians, 13 family physicians and 14 midwives; 18% were male and 82% were female. A summary of participant demographic characteristics is presented in Table 2.

**Table 2:** Participant Demographic Characteristics

Demographic Characteristics	Obstetricians *n* = 12	Family Physicians *n* = 13	Midwives *n* = 14	Total *N* = 39
Gender				
Female	8	10	14	32 (82%)
Male	4	3	0	7 (18%)
Years Practiced				
Mean	14.6 (range 1–28)	15.2 (range 2–37)	10.1 (range 1–20)	13 (range 1–37)
Age in years				
20–29	0	0	1	1 (2.5%)
30–39	3	3	4	10 (25.6%)
40–49	4	5	7	16 (41.0%)
50–59	3	0	2	5 (12.8%)
60–69	0	3	0	3 (7.7%)
Undisclosed	2	2	0	4 (10.3%)
Community				
size				
< 20,000	0	5	1	6 (15.4%)
20,000 to	1	0	1	2 (5.1%)
99,999	8	8	12	28 (71.8%)
> 100,000	3	0	0	3 (7.7%)

All participants reported screening their patients to determine eligibility for a TOLAC using the SOGC VBAC Guidelines [9, 48]. The themes presented are focused on factors maternity care providers considered when counselling pregnant women who are eligible for a TOLAC based on these guidelines [9, 48]. For ease of presentation, the themes are organized under the following categories: clinical/patient, health system, and provider factors (see Table 3). However, the themes are thickly intertwined.

**Table 3:** Factors that influence maternity care providers when counselling women on a TOLAC or PRCS

Factors	Themes
Clinical/patient factors	1. Women’s choice … with conditions 2. Provider perceptions of women’s risk tolerance 3. Provider assessment of potential for TOLAC success 4. Provider perceptions of women’s beliefs and attitudes about childbirth
Health care system factors	1. Colleague and facility support for TOLAC 2. Time to emergency CS
Provider factors	1. Provider preference for a PRCS or TOLAC 2. Scope of practice 3. Financial incentives and convenience related to PRCS 4. Past experiences with TOLAC and PRCS 5. Providers’ perspectives on risk of TOLAC

### Clinical/ patient factors

Providers considered multiple patient factors, including the woman’s preferred delivery method, clinical factors affecting a women’s chance for a successful TOLAC, women’s risk tolerance, and psychosocial/emotional factors.

### Women’s choice … with conditions

All providers who participated in the study shared that one of the most important factors considered when counselling was the woman’s preferred delivery method. They strongly voiced their belief that method of delivery was the woman’s ‘choice’. An obstetrician shared: *“I think what I want is less important than what she wants”* (OB14). A midwife stressed: “*It’s about supporting women to make choices that are right for them”* (MW11).

Interestingly, for some maternity care providers, support for eligible women’s choice for a TOLAC came with conditions. Most providers, particularly family physicians and obstetricians, stipulated that their support for women’s choice and specifically the choice of a TOLAC was dependent on the ‘condition’ that women were making, what providers considered, a ‘good’ choice. For providers, good choice was related to providers’ belief that TOLAC would have a high probability of being successful and not lead to risky complications. One obstetrician explained: *“It’s ultimately a woman’s choice no matter what, but when you start having the cards stacked against her, so when the baby is over the ninety fifth percentile, she hasn’t spontaneously [gone into labour], she’s going to get an infection, kind of counselling her … it’s a new conversation about elective Caesarean section versus VBAC* “(OB6). Some providers shared that if they believed a patient was making a ‘bad’ choice, they would try to dissuade them. As one obstetrician remarked: “*If I get the sense that a patient has a particular preference right off the top, I don’t work very hard to convince them otherwise unless when I weigh the risk of badness …*. I *don’t think they have an accurate sense of that*” (OB5).

Providers’ assessment of a ‘riskier’ choice was generally focused on a woman’s choice to have a TOLAC. None of the providers mentioned the risk of uterine rupture as the reason for their assessment of a ‘riskier’ choice. Furthermore, only one obstetrician, who had had a maternal death after a PRCS, identified PRCS as a potential ‘riskier’ choice.

All participants discussed the importance of their patients making an ‘informed choice’ about method of delivery. Most of the midwives and physicians stressed that they needed to provide their patients with a ‘balanced’ perspective of the risks and benefits associated with both TOLAC and PRCS. An obstetrician observed*:” I see it as my job to provide her with the best information and let her make the choice that is best for her*” *(OB14).* One midwife commented: “*We kind of give them as much information as we can, from as many sources as we can. One of my favorites is that Royal College of Obstetricians and Gynecologists paper, because it is very balanced. It doesn’t just show the risks of VBAC, it talks about the risks of … an elective repeat caesarean as well*” (MW01). Several physicians put less emphasis on the need for women to understand the risks associated with PRCS compared to TOLAC. This was evident in some physicians’ recommendation for PRCS when they believed their patients did not and/or could not understand the risks of TOLAC. PRCS, for some providers, was perceived as the less risky choice. One obstetrician reasoned: *“The lowest risk delivery is a successful vaginal delivery. The highest risk delivery is a failed vaginal delivery and CS in labour. So, the middle ground risk procedure is an elective CS”* (OB3).

Although all participants spoke about the need to counsel women on the risks and benefits of TOLAC and PRCS, several obstetricians and family physicians viewed counselling about TOLAC as more complicated than counselling about PRCS. Counselling on TOLAC was perceived to require additional time, without remuneration, and more teaching resources when compared to counselling about PRCS. One obstetrician explained: *“It’s sometimes hard to have enough time to counsel them [patients] appropriately, I mean there is no billing code [for payment] for counselling someone about VBAC”* (OB4). A family physician pointed out: “*There are not really any good resources to help people understand* [the risks and benefits of TOLAC]” (FP17). Some participants reported developing their own resources or using hospital developed resources to educate women about TOLAC and PRCS. Several care providers shared that when there was not enough time and resources to ‘completely’ inform a woman about TOLAC, they would recommend a PRCS.

Several physicians identified that some patients wanting TOLAC did not want to be informed about risks. An obstetrician noted: *“Sometimes I’ve had clients who … don’t want to hear about the risks”* (OB6). Patients’ capacity to grasp the risks of TOLAC due to a language barrier or limited comprehension were considerations. Participants shared comments such as, “*Most of my clients are low in economic circumstances. [The] information … covered they don’t understand* “(FP13); *“There are a number of patients … .who do not understand English…so it is hard to make them understand*” (FP16); and “*Wanting to make sure the patients understand … You say you could have your uterus rupture and it’s another thing that they understand what that means for themselves or their baby”* (OB6).

Midwives felt they had time for counselling on TOLAC. One midwife commented: “*We have time in our appointments to build relationships and we do discuss things with people*” (MW11). They identified that the majority of their patients with previous CS wanted TOLAC and choose midwifery care believing that a midwife was more likely to make a desired vaginal birth a reality. Some midwives found this belief challenging, particularly when patients, who had been refused a TOLAC by physicians based on sound medical advice, assumed midwives could make a vaginal birth happen. A midwife explained: “*I have clients who come to midwifery care and … have this expectation that magically we will make their birth experience go different from the previous one, that they will be protected from negative outcomes*” (MW29).

### Provider perception of Women’s risk tolerance

Many participants believed women needed to have a higher degree of risk tolerance to choose a TOLAC compared to a PRCS. Thus, assessing how much risk their patients would tolerate was an important consideration during counselling. An obstetrician noted*:”* Every *woman has a different level of risk they are willing to tolerate. As I go through things with her … I start to get a little bit of a sense where she would draw the line”* (OB14). “*There are patients [for whom] any risk is unacceptable. They tend not to VBAC. Other patients seem very risk tolerant, and so they choose to VBAC*” (OB15). Many participants shared that patients would choose a PRCS if they thought the baby would be at increased risk with a TOLAC. One obstetrician stated: “*Risk for the baby tends to be the highest consideration, and then secondary is their own personal risk … they hear about fetal complications related to uterine rupture even though those risks are minute … they’ll choose PRCS*” (OB6). Midwives also explored women’s risk tolerance: *“Some people feel like ‘I want every t crossed … I’m not comfortable with risk’ … .and then I have other people who feel, ‘I trust my body’”* (MW26). The risks discussed during counselling focused mostly on uterine rupture and immediate risks for the infant and surgical risks for the mother.

### Provider assessment of potential for TOLAC success

Most participants considered eligible patients’ chance for TOLAC success when counselling. A family physician noted: *“I usually review that there are ways that we can estimate as best we can their likelihood of success”* (FP23). Some obstetricians reported using specific TOLAC success predictor tools. As one obstetrician shared: “*I would usually quote them a risk of that [TOLAC success] based on using a VBAC chance of success calculator that I have linked to at some university in the US. Based on their age, the reason for their first CS, their BMI and their ethnicity, it spits out their chance of success*” (OB15).

### Provider perceptions of Women’s beliefs and attitudes about childbirth

Many participants shared that during counselling they considered how their patients felt about giving birth and past childbirth experiences. Some participants stated considering the potential negative psychological impact of the previous labour that led to a CS on their patients. A midwife commented: *“If she had a long awful labour that went nowhere … moved to CS, there can be a lot of feeling of failure wrapped up in that”* (MW55). A family physician similarly remarked: *“Women who have CSs they didn’t want … end up feeling like they somehow failed, that they are not true mothers … as stupid as that is, it is a very hard feeling to shake”* (FP20).

Many participants believed some women were afraid of childbirth, in that they feared labour, labour pain, pushing during vaginal birth, perineal trauma, and loss of control during labour and delivery. One family physician explained: “*I think women often are very scared about labour, and they are scared about vaginal and perineal trauma. I think that’s huge … that’s why a lot of women would want a CS* … *I want to get a sense of, is the woman, looking at me like*; ‘*there’s no way I’m going through that again’* [labour ending in unplanned CS], *then it’s unlikely, they have to be on board* [for a TOLAC](FP19). Some providers believed that most women perceived PRCS as the better choice. A family physician noted: *“CS, seems people perceive it, rightly or wrongly, as less painful and more dignified*” (FP28). Many participants believed that women valued the efficacy associated with a PRCS. A midwife explained: *“Planning a CS has some advantages. They know when they are going to have the baby … have their childcare in place … arrive at the hospital well rested”* (MW30).

A few participants believed that women were strongly influenced by ‘the baby first’ culture. A family physician described this culture as follows: “*There is such a culture of … women … just sacrificing themselves … this embedded system that what’s good for women doesn’t really count, it’s really what’s good for the baby … .so women will do stuff that is not in their best interest … because they believe it is safer for their child. People who are against VBAC, that is their politics … they really feel strongly about the baby”* (FP20).

### Health care system factors

#### Colleague and facility support for TOLAC

Colleague support for TOLAC was an important consideration for obstetricians and family physicians, particularly in small and medium-sized community hospitals. A few providers discussed the lack of support from their hospital for TOLAC. An obstetrician shared: “*My hospital really doesn’t like to do VBACs. My argument is if we can’t do VBACS, then we really shouldn’t be doing any kind of labour. What happens if there is a prolapse or whatever else? They (the hospital) have turned a deaf ear”* (OB4).

Some participants were hesitant to promote the option of TOLAC because their colleagues did not support TOLAC. They did not want their patients to be disappointed if the on-call physician refused to do a TOLAC. Several family physicians and midwives discussed hospital policy that required patients who wanted a TOLAC to have a consultation with an obstetrician. They strategically referred their patients to obstetrician consultants they believed were supportive of TOLAC to increase the likelihood the patient would be permitted a TOLAC, even if a non-supportive obstetrician was on-call. Family physicians and midwives also used this strategy even when the hospital policy did not require an obstetrical consult for TOLAC. A family physician explained: “*One of the things I look at is who the obstetrician who may be on call is … strategically what I will do is get a consultation ahead of time with an obstetrician who I know is supportive of VBAC … It can be very helpful to have a consultation on the chart when the patient goes into hospital, particularly if the obstetrician is someone who is unsupportive … so it is helpful for them to see that one of their colleagues was* [supportive of TOLAC]” (FP12).

#### Time to emergency CS

Several family physicians and some obstetricians, particularly those working in smaller communities, identified that they considered their hospital’s capacity to mount a timely emergency CS when counselling their patients on TOLAC. One family physician explained: “*I review the parameters of our institution, where we are a level one facility and we therefore have less resources … and in particular in mounting, in the speed we can mount a crash section [CS] for our worst fear, uterine rupture*” (FP15). Physicians in these facilities shared that they were careful when identifying patients for whom they would advise a TOLAC in their setting. One family physician commented: “*I’m very supportive of VBAC, but also very careful about selecting the right candidates*” (FP22). This approach meant that they looked beyond the TOLAC eligibility criteria set out by the SOGC to factors that might put the patient at higher risk for an unsuccessful TOLAC and/or uterine rupture. They sent these patients (who wanted TOLAC) to maternity care providers at institutions where emergency CS was readily available. As a family physician recounted: *“If somebody had a CS 20 months ago, we won’t do them because we are a level one [low risk maternity care centre]. We will refer them to some docs in X, which is a level II hospital”* (FP23). None of the midwives interviewed mentioned time to mount an emergency CS as a consideration. Some midwives commented that if patients felt strongly, they wanted a TOLAC at home, although this was not the midwives’ preference, they would support the choice. They felt it was the woman’s choice as long as the woman was informed about the risks of TOLAC and made an informed decision.

### Provider factors

#### Provider preferences for a PRCS or TOLAC

All participants identified method of delivery as the patient’s choice, and many expressed that they tried hard not to allow their own preferences for PRCS or TOLAC to influence their counselling. Some participants reported that they were not totally able to keep their own preferences from influencing their counselling. Within all three provider groups, preference or lack of preference for PRCS or TOLAC varied. For example, there were obstetricians who preferred TOLAC over PRCS or had no preference, as reflected in the following comments: “*I’ve always preferred a trial of labour. I’ve never been a supporter of elective CS. There are a number of reasons why a TOLAC is better*” (OB10). “*I’m pretty comfortable with both. I do lots of sections and our facility actually has a fairly high TOLAC rate”* (OB7). Providers’ personal delivery preferences were informed by several factors, including scope of practice, best practice guidelines on TOLAC, past experiences with TOLAC and PRCS, practice incentives, and their own risk tolerance during childbirth. One family physician commented: “*Some people are cutters and some people are non-cutters*” (FP20).

#### Scope of practice

The majority of midwives and family physicians interviewed preferred TOLAC. Their preference for TOLAC was partly based on the fact that their scope of practice did not include CS. A family physician noted: “*I’m a family doctor, a CS is never good for me, just selfishly, right? It’s no easier for me. CS is never to my advantage*” (FP20) Obstetricians, on the other hand, do both CS and TOLAC. One obstetrician shared his opinion: “*We as providers [obstetricians] are much happier to do a CS. Because you know for an obstetrician there is absolutely no reason not to be doing CS*” (OB4). Another obstetrician explained: “*A CS is no big deal*” (OB3).

#### Financial incentives and convenience related to PRCS

A few obstetricians disclosed that some of the reasons for their personal preferences for PRCS were scheduling convenience and financial benefit. One obstetrician explained: “*Economically you get more money. It’s an hour, it’s done, it’s scheduled. You don’t have to get up in the middle of the night most of the time. … The ideal way for an obstetrician would be to deliver everybody by CS, schedule them for a CS at 38 weeks gestation and just bang them out, five a day or however many a day and then you wouldn’t have to get up at night*” (OB4). One of the midwives shared similar thoughts about the convenience of PRCS and noted: “*There’s the whole efficiency and planning and certainly* [it is] *more efficient to book OR* [operating room] *time than it is to have unpredictable labour hours and staffing*” (MW50).

#### Past experiences with TOLAC and PRCS

Past experiences with TOLAC and PRCS influenced many participants’ preferred delivery method. Several obstetricians developed their preferences for TOLAC or PRCS based on previous experiences with emergency situations and poor maternal and perinatal outcomes related to delivery method. One participant, who preferred TOLAC, tearfully recounted a maternal death due to complications of an elective repeat CS: “*So my only* [maternal] *death was somebody who refused a TOLAC and had a CS”* (OB4). Another participant preferred PRCS because “*we’ve had some bad outcomes … [It’s] the most important factor in why I would prefer a PRCS to labouring”* (OB9).

Most family physicians spoke about having positive experiences with TOLAC. One family physician shared: *“I’ve had some fantastically wonderful VBACs that made me believe in the whole process*” (FP17). The family physicians who tended to prefer PRCSs had experienced a uterine rupture in their practice. One family physician noted: “[uterine rupture] *kind of influences you a bit. … I’ve probably seen more positive experiences around elective repeat CS”* (FP21)*.* None of the midwives reported having had any bad experiences with TOLAC or PRCS.

#### Providers’ perspectives on risk of TOLAC

The majority of obstetricians observed that failed TOLAC is associated with increased risk for poorer maternal and perinatal outcomes than PRCS. They expressed fear of the catastrophic event, i.e. ‘uterine rupture’. Fear of uterine rupture was the major factor many obstetricians stated their practice preference was PRCS. One obstetrician explained: “*There is definitely a danger. We can’t predict who is going to rupture and who is not. We just know the statistic is one in one thousand. Which means if I do a thousand VBACs I am going to have one catastrophic rupture. It could be tomorrow morning … That’s reality. That’s going to be a dead baby and a uterus that has to be removed. The outcome is so awful that one has to consider whether you do a few sections and to save that VBAC rupture … I’m one of the guys who does them [TOLAC] but I’m always anxious about the lady in labour*” (OB5). Another obstetrician summated: “*A ruptured uterus during labour is a big deal. Why would anyone risk a rupture during labour given a CS is no big deal?”* (OB15).

The midwives interviewed had a different perspective on the risks of TOLAC. One midwife shared: “*When it comes to VBAC, there are higher risks, so you have further conversations. But reality is statistically [speaking] the vast majority of people will have success with their VBAC attempts, so why are we pathologizing them?”* (MW26).

## Discussion

This is one of the first Canadian studies to examine factors maternity care providers consider when counselling pregnant women, eligible for TOLAC, on mode of childbirth. The range of clinical/patient, health care system and provider factors that emerged are intricately intertwined influencing providers’ counsel.

All maternity care providers in our study identified that delivery method was a woman’s choice and that their role was to counsel women about the risks and benefits of various options. Participants implicitly espoused the informed choice and shared decision-making model promoted by Canada’s Guidelines on Family-Centered Maternity Care, the SOGC, and Canadian Association of Midwives [9, 49–51]. Likewise, in previous Canadian and Australian studies, providers supported shared decision-making [37, 52]. However, our findings are a stark contrast to results of qualitative studies done with maternity care providers in Sweden, Finland, and the Netherlands where TOLAC rates are much higher than in Canada [29, 53]. Obstetricians and midwives in these studies had a strong belief in ‘normal birth’ and TOLAC was their first choice for eligible women [29, 53]. Lundgren and colleagues, in their 2015 study of Swedish, Finish and Dutch maternity care providers, reported that they expressed concern that if women were given the choice of TOLAC or PRCS, the rates of PRCS would increase [29] and, similar to a 2018 Swedish study [53], they found that obstetricians made the final decisions on delivery method [29]. If they are correct in their belief that women, if given the choice, would choose PRCS, women may be one of the main drivers of the low rates of TOLAC in Canada. However a move away from a shared patient-provider decision-making model in Canada is likely an unacceptable strategy to increase TOLAC rates for cultural, legal, and ethical reasons [54]. Additionally, the evidence remains unclear whether TOLAC or PRCS leads to the best maternal and infant outcomes [21, 55].

Interestingly, many of our participants put conditions on eligible women’s ‘choice’ of TOLAC, including that women needed to be fully informed and understand the risks of TOLAC. These participants did not place the same condition on women’s ‘choice’ of PRCS. In fact, several participants identified PRCS as the default method of delivery when women did not understand the risks associated with TOLAC. As in previous studies (Panda et al., 2018), our findings reflect that some providers had normalized and preferred PRCS.

Several barriers to counselling women, particularly related to TOLAC emerged in our study. Lack of time was a common complaint among physicians. This was not the case in Munro and colleagues’ study in British Columbia who found that providers were motivated to spend time to discuss delivery options with patients [37]. Our findings may relate to participants’ limited access to quality patient TOLAC resources and lack of reimbursement for counselling about TOLAC. Ontario obstetricians and many family physicians are paid through the government’s fee for service plan. Patient counselling, specifically about TOLAC, is not identified in the payment schedule, while counselling in general is covered for prenatal care [56]. This suggests that some providers viewed TOLAC counselling as an extra service beyond general prenatal counselling. Interestingly, these providers did not identify the same issue with regard to counselling about PRCS. Patient factors such as language barriers and/or low education were also identified as challenges in counselling women about TOLAC. When women could not understand the risk of TOLAC, most participants recommended a PRCS. This practice may partly explain the higher rates of CS among women born outside Canada [57, 58]. Language barriers are common among immigrants using health services in Canada [59, 60]. Over 29% of Ontario’s population is born outside Canada [61].

Many study participants described a ‘highly risk oriented’ approach to counselling about TOLAC compared to PRCS. This approach is concerning as maternity care providers’ advice is a major influence on women’s choice of delivery method [18, 23, 62]. Counselling that is highly ‘risk oriented’ and not balanced regarding the risks of TOLAC and PRCS can produce fear and eliminate trust in women trying to make the best choice for themselves [28, 63, 64]. Furthermore, a ‘highly risk oriented’ approach to TOLAC may inappropriately lower women’s risk tolerance [65], a factor many participants reported considering during counselling. Studies of counselling approaches in countries with high TOLAC rates report that obstetricians and midwives were supportive of TOLAC, considered VBAC as the first alternative, felt confident about TOLAC, and believed they needed to help women build trust in VBAC [22, 29]. Moreover, women reported that receiving information from supportive clinicians, knowing the advantages of VBAC and viewing VBAC as the first alternative for themselves and their providers when there were no complications, supported VBAC [28]. Although some of our study participants were very comfortable with TOLAC, none identified VBAC as the first alternative. Many were most comfortable with PRCS as the first alternative and were more likely to promote PRCS over TOLAC. No study participants discussed the need to build women’s confidence in TOLAC. Their focus was on information sharing and helping women understand the information.

The ability to predict a woman’s chance of successful VBAC is important to providers when counselling on delivery method. Providers in our study considered factors that are commonly associated with TOLAC success, such as previous vaginal delivery, and decreased TOLAC success, such as history of obstructed labour. Some participants used tools, such as the Maternal Fetal Medicine Unit (MFMU) VBAC calculator [66] to predict odds of TOLAC success. However, recent studies evaluating factors commonly associated with decreased TOLAC success, such as primary CS for arrest of descent, failure to progress in labour and no previous vaginal delivery, have reported higher than expected VBAC success rates ranging from 49 to 73% [67–70]. Furthermore, tools used to predict VBAC success lack demonstrated validity and reliability. For example, the MFMU calculator [66], a widely used tool, has demonstrated good predictability for women who have a high chance of TOLAC success, but underestimates VBAC success in women who have moderate to low predicted success [71, 72].

Not surprisingly, access to emergency CS in the event of uterine rupture was an important health service factor participants considered when counselling about TOLAC [9, 37], and particularly among those who worked in small and medium-sized community hospitals where anesthesiologists are often called in from home [73]. They cautiously recommended TOLAC in these facilities, and sometimes recommended women deliver in hospitals with better access to timely emergency CS. This finding may partly explain the regional variation in repeat CS across Ontario, ranging from 69.8 to 88.2% [2], and why VBAC rates are higher in hospitals where emergency CS are readily available [74, 75]. Interestingly, Munro and colleagues [37] found that providers in two rural British Columbia, Canada communities believed that they provided safe access to emergency CS even though surgical staff were not always on site. To date there is only fair quality evidence and expert opinion to support the need for on-site surgical backup to ensure patient safety during TOLAC [21].

Lack of support from colleagues and hospitals has been previously identified as a barrier to TOLAC [22]. Similarly, we found that some participants had colleagues who did not support TOLAC. They were hesitant to offer women a TOLAC when they knew the on-call physicians would not support this option. Reasons for lack of provider support for TOLAC is not well understood within the Canadian context. In the United States, despite the evidence supporting the safety of TOLAC for eligible women [3, 7, 76], many hospitals and providers are unsupportive [22, 64, 77–80]. Reasons given for non-support of TOLAC include unsupportive hospitals, public vs. private insurance, lack of obstetricians, obstetricians’ preferences, and lack of anesthesiologists [22, 77, 80, 81]. Additionally, provider liability is often identified as a reason for lack of TOLAC support [22]. Participants in our study denied being concerned about personal liability. Professional VBAC guidelines may also contribute to the lack of provider and hospital support for TOLAC. Triebwasser and colleagues [80] identified that the American College of Obstetricians and Gynecologists’ (ACOG) VBAC guidelines [82] requiring ‘immediate’ availability of emergency CS was an important factor in some United States hospitals’ refusal to allow TOLAC. The SOGC’s new 2019 VBAC guidelines [21] recommends ‘immediate’ availability of CS, a change from previous wording, i.e., ‘timely’ access, which was often interpreted as within 30 min [9, 48]. The recommendation for ‘immediate’ CS availability, based on fair quality evidence and expert opinion [21], may decrease the number of colleagues who support TOLAC and may result in some hospitals adopting unsupportive TOLAC policies as seen in the United States [77].

Several providers in our study, as in previous studies [22, 31], believed that PRCS is safer than TOLAC for eligible women. Participants’ cognitive bias, assuming PRCS is safer than TOLAC, was founded on personal bad experiences with TOLAC. While understandable on a personal emotive level, this bias is counterproductive in health care and professionally as it does not take into account best evidence [83]. Perhaps it is because best evidence regarding the safety of PRCS and TOLAC is most apparent when examined at the population level and less evident at the individual patient level [24, 84]. Provider psychological traits may partly explain the cognitive bias some participants had about TOLAC. In a large American study, Yee and colleagues [85] examined the relationship of obstetricians’ psychological traits and delivery outcomes among women with a prior CS. They found that obstetricians with high proactive coping and low anxiety had the highest TOLAC success rates [85].

Convenience and financial gain are often reported as reasons maternity care providers prefer PRCS to TOLAC [22]. Only a small number of study participants identified they preferred PRCS for these reasons. Payment for CS in Ontario is very similar to payment for vaginal delivery [56]. For the small number of providers who favored PRCS for convenience and pecuniary reasons, it was the daytime scheduling of deliveries and the ability to do several planned CS in a day that made PRCS more appealing.

### Strength and limitations

A strength of the study is the use of maximum variation sampling whereby we interviewed participants from Ontario’s three maternity care provider groups across rural and urban regions and who practiced in low risk to complex maternity care settings. This approach allowed us to develop a comprehensive description of a diverse range of factors providers considered during counselling. A comprehensive understanding was further supported by our continued recruitment until we achieved data saturation. Study limitations include that the views of maternity care providers in this study may not reflect the perspectives of all maternity care providers across Canada as only those who practiced in Ontario were interviewed. Additionally, there was potential selection bias in that those with an interest in the topic might have been more likely to participate than those with little interest, and a social desirability bias may have influenced what respondents conveyed.

## Conclusion

Maternity care providers strived to support women in their choice of delivery method. Many attempted to provide a balanced perspective of the risks and benefits of both TOLAC and PRCS but some lacked resources and time for counselling. Several providers perceived TOLAC as riskier than PRCS and were more likely to support a PRCS if they felt women did not understand the risks of TOLAC. Research from countries with high TOLAC/VBAC rates indicate that TOLAC being the providers’ first choice and building women’s confidence in TOLAC may be key to TOLAC uptake [28, 30, 64]. This perspective was not evident among providers in our study. If the rate of VBAC is to be increased in Canada, provider barriers to the promotion of TOLAC need to be addressed. Interventions to foster a paradigm shift where TOLAC and PRCS are equally considered, not only among maternity care providers but also in society, may help decrease PRCS rates. Strategies such as nationally developed evidence-based patient education resources on TOLAC and PRCS, continuing education on risks and benefits of TOLAC and PRCS for maternity care providers, and provincially developed TOLAC hospital policies tailored to individual hospital maternity care capacity may help increase TOLAC rates. Additionally, more research is required to understand the complex reasons for the high uptake of PRCS when TOLAC is an option, and to develop valid and reliable tools to evaluate women’s probability of successful VBAC. Furthermore, interventions need to be developed and evaluated to promote TOLAC uptake among eligible women, not only at the provider level, but also at patient, hospital, and societal levels. Finally, more rigorous research should be undertaken to determine the surgical and anesthesia capacity needed to support patient safety in the rare event of uterine rupture.

## Data Availability

We do not have consent from participants, or the research ethics boards to share the data collected in our study. Specific inquiries about the data can be sent to Dr. Christine Kurtz Landy.

## Abbreviations

CS: Caesarean Section
OECD: Organization for Economic Co-operation and Development
TOLAC: Trial of labour after caesarean section
PRCS: Planned repeat caesarean section
VBAC: Vaginal Birth after Caesarean Section
SOGC: Society of Obstetricians and Gynecologists of Canada
ACOG: American College of Obstetricians and Gynecologist

## References

[1] OECD. Health at a glance 2019: OECD; 2019. 10.1787/4dd50c09-en.

[2] CIHI. Quick Stats Metadata: Childbirth Indicators by Place of Residence CB1 2017–2018. Ottawa; 2019. https://apps.cihi.ca/mstrapp/asp/Main.aspx? Server=apmstrextprd_i&project=Quick Stats&uid=pce_pub_en&pwd=&evt=2048001&visualizationMode=0&documentID=029DB170438205.

[3] Guise J-M, Denman MA, Emeis C, Marshall N, Walker M, Fu R (2010). Vaginal birth after cesarean: new insights on maternal and neonatal outcomes. Obstet Gynecol.

[4] Macfarlane A, Blondel B, Mohangoo A, Cuttini M, Nijhuis J, Novak Z (2016). Wide differences in mode of delivery within Europe: risk-stratified analyses of aggregated routine data from the euro-Peristat study. BJOG An Int J Obstet Gynaecol.

[5] Martin J, Hamilton BE, MJK O (2019). Births in the USA: NCHS Data Brief No.

[6] Rossignol M, Moutquin JM, Boughrassa F (2013). Preventable obstetrical interventions: how many caesarean sections can be prevented in Canada?. J Obstet Gynaecol Can.

[7] ACOG (2019). ACOG Practice Bulletin Summary: Vaginal Birth adter Cesarean Delivery. Ostet Gynecol.

[8] Guise JM, Eden K, Emeis C, Denman MA, Marshall N, Fu RR (2010). Vaginal birth after cesarean: new insights.

[9] Martel M-J, MacKinnon CJ (2018). No. 155-guidelines for vaginal birth after previous caesarean birth. J Obstet Gynaecol Can.

[10] American College of Obstetricians and Gynecologists A (2019). ACOG practice bulletin no. 205: Vaginal birth after cesarean delivery. Obstet Gynecol.

[11] Royal College of Obstetricians and Gynaecologists (2015). Birth after previous caesarean birth. R Coll Obstet Gynaecol.

[12] Fobelets M, Beeckman K, Faron G, Daly D, Begley C, Putman K (2018). Vaginal birth after caesarean versus elective repeat caesarean delivery after one previous caesarean section: a cost-effectiveness analysis in four European countries. BMC Pregnancy Childbirth.

[13] Zhao Y, Zhang J, Zamora J, Vogel JP, Souza JP, Jayaratne K (2017). Increases in caesarean delivery rates and change of perinatal outcomes in low- and middle-income countries: a hospital-level analysis of two WHO surveys. Paediatr Perinat Epidemiol.

[14] Smithies M, Woolcott CG, Brock J-AK, Maguire B, Allen VM (2018). Factors associated with trial of labour and mode of delivery in Robson Group 5: a select group of women with previous caesarean section. J Obstet Gynaecol Can.

[15] Soltsman S, Perlitz Y, Ben Ami M, Ben SI (2018). Uterine rupture after previous low segment transverse cesarean is rarely catastrophic. J Matern Neonatal Med.

[16] Stattmiller S, Lavecchia M, Czuzoj-Shulman N, Spence AR, Abenhaim HA (2016). Trial of labor after cesarean in the low-risk obstetric population: a retrospective nationwide cohort study. J Perinatol.

[17] Flamm B (1997). Vaginal birth after cesarean delivery: an admission scoring system. Obstet Gynecol.

[18] Ryan GA, Nicholson SM, Morrison JJ (2018). Vaginal birth after caesarean section: current status and where to from here?. Eur J Obstet Gynecol Reprod Biol.

[19] Scott JR (1997). Avoiding labor problems during vaginal birth after cesarean delivery. Clin Obstet Gynecol.

[20] Weinstein D, Benshushan A, Ezra Y, Rojansky N (1996). Vaginal birth after cesarean section: current opinion. Int J Gynaecol Obstet.

[21] Dy J, DeMeester S, Lipworth H, Barrett J (2019). No. 382-trial of labour after caesarean. J Obstet Gynaecol Can.

[22] Panda S, Begley C, Daly D (2018). Clinicians’ views of factors influencing decision-making or caesarean section: a systematic review and metasynthesis of qualitative, quantitative and mixed methods studies. PLoS One.

[23] Metz TD, Stoddard GJ, Henry E, Jackson M, Holmgren C, Esplin S (2013). How do good candidates for trial of labor after cesarean (TOLAC) who undergo elective repeat cesarean differ from those who choose TOLAC?. Am J Obstet Gynecol.

[24] Black M, Entwistle VA, Bhattacharya S, Gillies K (2016). Vaginal birth after caesarean section: why is uptake so low? Insights from a meta-ethnographic synthesis of women’s accounts of their birth choices. BMJ Open.

[25] Chen S-W, Hutchinson AM, Nagle C, Bucknall TK (2018). Women’s decision-making processes and the influences on their mode of birth following a previous caesarean section in Taiwan: a qualitative study. BMC Pregnancy Childbirth.

[26] Eden KB, Hashima JN, Osterweil P, Nygren P, Guise J-M (2004). Childbirth preferences after cesarean birth: a review of the evidence. Birth..

[27] Kaimal AJ, Kuppermann M (2010). Understanding risk, patient and provider preferences, and obstetrical decision making: approach to delivery after cesarean. Semin Perinatol.

[28] Nilsson C, van Limbeek E, Vehvilainen-Julkunen K, Lundgren I (2017). Vaginal birth after cesarean. Qual Health Res.

[29] Lundgren I, van Limbeek E, Vehvilainen-Julkunen K, Nilsson C (2015). Clinicians’ views of factors of importance for improving the rate of VBAC (vaginal birth after caesarean section): a qualitative study from countries with high VBAC rates. BMC Pregnancy Childbirth.

[30] Lundgren I, Healy P, Carroll M, Begley C, Matterne A, Gross MM (2016). Clinicians’ views of factors of importance for improving the rate of VBAC (vaginal birth after caesarean section): a study from countries with low VBAC rates. BMC Pregnancy Childbirth.

[31] Cox KJ (2011). Providers’ perspectives on the vaginal birth after cesarean guidelines in Florida, United States: a qualitative study. BMC Pregnancy Childbirth.

[32] Kamal P, Dixon-Woods M, Kurinczuk JJ, Opeenheimer C, Squire P, Waugh J, Kamal P, Dixon-Woods M, Kurinczuk JJ, Oppenheimer C (2005). Squire P WJ. Factors influencing repeat caesarean section: qualitative exploratory study of obstetricians and midwives’ accounts. BJOG..

[33] Sur S, Murphy KWMI (2009). Delivery after caesarean section: consultant obstetricians’ professional advice and personal preferences. J Obstet Gynaecol (Lahore).

[34] Young CB, Lui S, Muraca GM (2018). Mode of Delivery after previous Cesarean birth, and associated maternal and neonatal morbidity. Can Med Assoc J.

[35] Klein MC, Kaczorowski J, Tomkinson J, Hearps S, Baradaran N, Brant R (2011). Family physicians who provide intrapartum care and those who do not: very different ways of viewing childbirth. Can Fam Physician.

[36] Klein MC, Liston R, Fraser WD, Baradaran N, Hearps SJC, Tomkinson J (2011). Attitudes of the new generation of Canadian obstetricians: how do they differ from their predecessors?. Birth..

[37] Munro S, Kornelson J, Corbett K, Wilcox E, Bansback N, Janssen P (2017). Do women have a choice? Care providers’ and decision makers perspectives on barriers to access of health services for birth after a previous cesarean. Birth Issues Perinat Care.

[38] Sandelowski M (2000). Whatever happened to qualitative description?. Res Nurs Health.

[39] Canada S (2018). Table 17-10-0008-01 estimates of the components of demographic growth, annual.

[40] BORN. One in a million. BORN Ontario biennial report 2016-2018. Ottawa. p. 2018. https://www.bornontario.ca/en/publications/resources/Documents/BORN-Biennial-Report-Feb-2019.pdf.

[41] Patton MQ (2002). Qualitative Research & Evaluation Methods.

[42] Hsieh H-F, Shannon SE (2005). Three approaches to qualitative content analysis. Qual Health Res.

[43] Miles MB, Huberman, AM. An expanded sourcebook: Qualitative data analysis, 2nd ed. Thousand Oaks: Sage; 1994.

[44] QSR (2014). NVivo 10 research software for analysis and insight.

[45] Hewitt-Taylor J (2001). Use of constant comparative analysis in qualitative research. Nurs Stand.

[46] Creswell JW, Miller DL (2000). Determining validity in qualitative inquiry. Theory Pract.

[47] Krefting L (1991). Rigor in qualitative research: the assessment of trustworthiness. Am J Occup Ther.

[48] Lalonde AB (2005). Guidelines for vaginal birth after previous caesarean birth. Int J Gynecol Obstet.

[49] Public Health Agency of Canada. Family-centred maternity and newborn care: National guidelines. Ottawa; 2018. https://www.canada.ca/en/public-health/services/maternity-newborn-care-guidelines.html.

[50] Stirling D, Vanbesien R, McDougall R (2017). Informed decision making in labour and birth.

[51] Stirling D, Vanbesien J, Mcdougall R. Informed decision-making for labour & birth www.opha.on.ca. Accessed 13 Nov 2018.

[52] Bryant J, Porter M, Tracy SK, Sullivan EA (2007). Caesarean birth: consumption, safety, order, and good mothering. Soc Sci Med.

[53] Panda S, Daly D, Begley C, Karlström A, Larsson B, Bäck L (2018). Factors influencing decision-making for caesarean section in Sweden – a qualitative study. BMC Pregnancy Childbirth.

[54] Dexter SC, Windsor S, Watkinson SJ (2014). Meeting the challenge of maternal choice in mode of delivery with vaginal birth after caesarean section: a medical, legal and ethical commentary. BJOG.

[55] Dodd JM, Crowther CA, Huertas E, Guise JM, Horey D. Planned elective repeat caesarean section versus planned vaginal birth for women with a previous caesarean birth. Cochrane Database Syst Rev. 2013. 10.1002/14651858.CD004224.pub3. 10.1002/14651858.CD004224.pub2 15495090

[56] Ministry of Health and Long Term Care O (2015). Physician services under the Health Insurance Act. The Schedule of Benefits: Physician Services, Regulation 552 of the Health Insurance Act.

[57] Gagnon AJ, Van Hulst A, Merry L, George A, Saucier JF, Stanger E, DE Wahoush OS (2013). Cesarean section rate differences by migration indicators. Arch Gynecol Obstet.

[58] Mumtaz Z, O’brien B, Higginbottom G, O’Brien B, Higginbottom G (2014). Navigating maternity health care: a survey of the Canadian prairie newcomer experience. BMC Pregnancy Childbirth.

[59] Kalich A, Heinemann LGS (2016). A scoping review of immigrant experience of health care access barriers in Canada. J Immigr Minor Health.

[60] Higginbottom G, Bell A, Arsenault J, Pillay J (2012). An integrative review of experiences of maternity services for immigrant women in Canada. Divers Heal Soc Care.

[61] Ministry of Finance O. 2016 Censsus Highlights: Factsheet 8 Immigration. Toronto; 2017. file:///Volumes/NO NAME/Qualitative provider paper/Immigrants in Ontario.pdf.

[62] Bonzon M, Gross MM, Karch A, Grylka-Baeschlin S (2017). Deciding on the mode of birth after a previous caesarean section – an online survey investigating women’s preferences in Western Switzerland. Midwifery..

[63] Keedle H, Schmied V, Burns E, Dahlen H (2018). The journey from pain to power: a meta-ethnography on women’s experiences of vaginal birth after caesarean. Women Birth.

[64] Lundgren I, Begley C, Gross MM, Bondas T (2012). “Groping through the fog”: a metasynthesis of women’s experiences on VBAC (vaginal birth after caesarean section). BMC Pregnancy Childbirth.

[65] Dahlen HG, Horner C (2013). Motherbirth or childbirth: a prospective study of vaginal birth after caesarean section blogs. Midwifery..

[66] Grobman W, Lai Y, Landon MB, Spong CY, Leveno KJ, Rouse DJ, Varner MW, Moawad AH, Caritis SN, Harper M, Wapner RJ, Sorokin Y, Miodovnik M, Carpenter M, O’Sullivan MJ, Sibai BM, Langer O, Thorp JM, Mercer BM. Development of a nomogram for predicition of vaginal delivery after cesarean delivery. Obstet Gynecol. 2007;109:806–12. 10.1097/01.AOG.0000259312.36053.02 17400840

[67] Ashwal E, Wertheimer A, Aviram A, Wiznitzer A, Yogev Y, Hiersch L (2016). Prediction of successful trial of labor after cesarean - the benefit of prior vaginal delivery. J Matern Neonatal Med.

[68] Fox NS, Namath AG, Ali M, Naqvi M, Gupta S, Rebarber A. Vaginal birth after a cesarean delivery for arrest of descent. J Matern Fetal Neonatal Med. 2018:1476–4954. 10.1080/14767058.2018.1443069. 10.1080/14767058.2018.1443069 29455594

[69] Lewkowitz AK, Nakagawa S, Thiet MP, Lewkowitz AK, Nakagawa S, Thiet MP, Rosenstein MG (2015). Effect of stage of initial labor dystocia on vaginal birth after cesarean success. Am J Obstet Gynecol.

[70] Place K, Kruit H, Tekay A, Heinonen S, Rahkonen L (2019). Success of trial of labor in women with a history of previous cesarean section for failed labor induction or labor dystocia: a retrospective cohort study. BMC Pregnancy Childbirth.

[71] Perez WM, Vricella LK, Gilad GA, Tomlinson TM (2019). 809: validity of the MFMU antenatal VBAC calculator in the contemporary labor management era. Am J Obstet Gynecol.

[72] Thornton P (2018). Limitations of vaginal birth after cesarean success prediction. J Midwifery Women’s Heal.

[73] Angle P, Kurtz Landy C, Murthy Y, Cino P (2009). Key issues and barriers to obstetrical anesthesia care in Ontario community hospitals, with fewer than 2000 deliveries annually. Can J Anesth.

[74] Lavender T, Hofmeyr GJ, Neilson JP, Kingdon C, Gyte GM. Caesarean section for non-medical reasons at term. Cochrane Database Syst Rev. 2012. 10.1002/14651858.CD004660.pub3. 10.1002/14651858.CD004660.pub2 16856054

[75] Triebwasser JE, Kamder NS, Langen ES, Basu T, Syrjamaki J, Thomason A, Smith RD, Morgan DM (2019). Hospital contribution to variation in rates of vaginal birth after cesarean. J Perinatol.

[76] Signore C, Spong CY (2010). Vaginal birth after cesarean: new insights manuscripts from an NIH consensus development conference, march 8-10, 2010. Semin Perinatol.

[77] Barger MK, Dunn JT, Bearman S, DeLain M, Gates E (2013). A survey of access to trial of labor in California hospitals in 2012. BMC Pregnancy Childbirth..

[78] Keedle H, Schmied V, Burns E, Dahlen HG (2015). Women’s reasons for, and experiences of, choosing a homebirth following a caesarean section. BMC Pregnancy Childbirth..

[79] Nilsson C, Lalor J, Begley C, Carroll M, Gross MM, Grylka-Baeschlin S (2017). Vaginal birth after caesarean: views of women from countries with low VBAC rates. Women Birth.

[80] Triebwasser JE, Kamdar NS, Langen ES, Moniz MH, Basu T, Syrjamaki J (2019). Hospital contribution to variation in rates of vaginal birth after cesarean. J Perinatol.

[81] Schifrin BS, Cohen WR (2013). The effect of malpractice claims on the use of caesarean section. Best Pract Res Clin Obstet Gynaecol.

[82] Gynecologists AC (2010). Of O and. Practice bulletin no.115: vaginal birth after previous cesarean delivery. Obstet Gynecol.

[83] Lyerly AD, Mitchell LM, Armstrong EM, Harris LH, Kukla R, Kuppermann M, Little MO (2009). Risk and the pregnant body. Hast Cent Rep.

[84] Fenwick J, Gamble J, Hauck Y (2006). Reframing birth: a consequence of cesarean section. J Adv Nurs.

[85] Yee LM, Liu LY, Grobman WA (2014). The relationship between obstetricians’ cognitive and affective traits and delivery outcomes. Am J Obstet Gynecol.

